# CKAP2 Regulated by TFDP1 Promotes Metastasis and Proliferation of Colorectal Cancer through Affecting the Tumor Microenvironment

**DOI:** 10.4014/jmb.2407.07008

**Published:** 2024-09-20

**Authors:** Zhiqiang Zhong, Shi Cheng, Yang Liu

**Affiliations:** Department of General Surgery, Beijing Tiantan Hospital, Capital Medical University, Beijing 100070, P.R. China

**Keywords:** CKAP2, colorectal cancer, tumor microenvironment, TFDP1, macrophage

## Abstract

The current pathological and physiological evaluation system for colorectal cancer (CRC) is limited; thus, effective biological targets to diagnose and treat this disease are urgently needed. In this study, we used qRT-PCR for detecting mRNA levels of genes. The levels of protein were identified by western blot, immunohistochemistry, and immunofluorescence assays. In addition, functional experiments were used to evaluate the role of cytoskeleton associated protein (CKAP) 2 in CRC cells and human umbilical vein endothelial cells (HUVECs). Bioinformatics analysis was employed to predict the binding relationship of CKAP2 and TFDP1, which was confirmed through dual luciferase reporter assay and immunoprecipitation assay. Furthermore, we injected human colorectal carcinoma HCT116 cells into mice flanks, and we injected Luciferase-labeled HCT116 cells into mice tail vein. HE staining was used to detect tumor nodules. As a result, high CKAP2 expression was found in CRC cells and tissues. CKAP2 silencing reduced CRC cell migration, invasion, proliferation, and epithelial-mesenchymal transition. Moreover, CKAP2 expression was positively associated with M2 macrophage levels. CKAP2 promoted protein expression of CD86, CD206, IL-1β, and CCL17. Moreover, CKAP2 promoted the proliferation of HUVECs and angiogenesis via affecting the tumor microenvironment (TME). We also found that CKAP2 could interact with TFDP1. The inhibitory impacts of TFDP1 downregulation on CRC cell’ proliferation, migration, and invasion were reversed via CKAP2 overexpression. In vivo silencing of CKAP2 repressed tumor growth and metastasis. Overall, CKAP2 was positively regulated by TFDP1, which promoted tumorigenesis and metastasis in CRC.

## Introduction

Colorectal cancer (CRC) is the 2^nd^ most common cause of cancer death [1-6]. Lymph node metastasis, distant organ metastasis, extraintestinal vascular invasion, and peripheral nerve invasion are high-risk factors for CRC [7-9]. The methods of tumor treatment are surgical resection, radiotherapy, chemotherapy, and immunotherapy. Of these, surgical resection is the main treatment option for patients with CRC having no distant metastasis [10, 11]. However, the current pathological and physiological evaluation system for CRC has some limitations and cannot be relied upon to accurately formulate appropriate treatment plans [12, 13].

CKAP2 is a protein associated with microtubule and mitotic spindle stability that can affect cell division [14-16]. CKAP2 expression is proved to be upregulated in diverse malignant tumors, such as gastric cancer, lung adenocarcinoma, and skin T-cell lymphoma [17-19]. CKAP2 protein expression is highest in the cell cycle’s G2/M phase and is closely related to proliferative activity [20]. The biological mechanisms of CKAP2 affecting the development of breast cancer [21, 22], gastric cancer [17], and ovarian cancer [23] have been probed, but the expression and role of CKAP2 in CRC remain unclear.

Here, we investigated CKAP2 protein expression level in CRC, and then we examined CKAP2’s impacts on proliferation and CRC lung metastasis. CKAP2 is a possible target in the treatment of CRC.

## Materials and Methods

### Cell Culture and Transfection

Human CRC cell lines (HT29, HCT116, SW480, LOVO, and SW620) and normal FHC cells were bought from American Type Culture Collection (ATCC, USA) and cultured in RPMI-1640 medium + 10% fetal bovine serum (FBS). Then, all cells were incubated in a condition of 37°C and 5% CO_2_.

Plasmids (RiboBio, China), including sh-CKAP2, sh-NC, pcDNA3.1-CKAP2, pcDNA3.1-NC, si-TFDP1, and si-NC were used. These plasmids were transfected into SW480 and HCT116 cells through Lipofectamine 3000 (Invitrogen, USA) and cultivated for 2 days. The transfection efficiency was detected via western blotting.

THP-1 cells from ATCC were induced with PMA for 2 days to obtain M0 macrophages. The induced THP-1 cells were preserved with the cell supernatant from HCT116/SW480 cells.

### qRT-PCR

Total RNAs from CRC cells and FHC cells were extracted by using the TRIzol reagent (Invitrogen). The reverse transcription kit (Invitrogen) was utilized to produce the complementary DNAs for mRNAs. Then, we conducted qRT-PCR using a SYBR Green qRT-PCR Kit (Promega, USA) for CKAP2. Finally, relative expression of CKAP2 was calculated using the 2^-ΔΔct^ method normalized to GAPDH [24] (See Table 1 for the primers).

### Western Blotting

HT29, HCT116, LOVO, SW480, SW620, and FHC cells were dissolved through RIPA buffer (Beyotime, China). Total proteins were purified and then quantified via bicinchonininc acid (BCA) protein kits (Thermo Fisher Scientific, China). After that, proteins were isolated through SDS-PAGE (10%) and transported to PVDF membranes. After being blocked with skimmed milk (5%), the proteins were incubated with anti-CKAP2 (ab85889, Abcam, USA, dilution: 1/2000), anti-E-cadherin (ab40772, dilution: 1/2000), anti-N-cadherin (ab76011, dilution: 1/2000), anti-TFDP1 (ab186831, dilution: 1/2000), and anti-β-actin (ab8227, dilution: 1/2000) for 1 day at 4°C. After washing of the primary antibodies, the proteins continued to incubate with the HRP secondary antibody (ab6721, dilution: 1/2000) for 1 h. Finally, protein bands were observed via the ECL chemiluminescent system and ImageJ was used for quantifying protein blots.

### CCK-8 Assay

The proliferative capabilities of SW480, HCT116 cells, and HUVECs were assessed through a CCK-8 kit (Beyotime). SW480 and HCT116 cells (1 × 10^3^/well) were plated in a 96-well plate, followed by incubation for 0 h, 24 h, 48 h, and 72 h. After that, CCK-8 reagent (10 μl) was added to wells at room temperature. The absorbance at 450 nm was monitored with a microplate reader (BMG Labtech, USA).

### EdU Assay

SW480 and HCT116 cells were plated in 96-well plates and mixed with EdU solution (RiboBio). After that, the cells were immobilized by formaldehyde and Triton X-100 and mixed with glycine. Next, the cells were mixed with 1×Apollo dye liquor (in the dark) and then stained by DAPI. Lastly, EdU-positive cells were counted under a fluorescence microscope.

### Transwell Assay

Transwell chambers (8.0 μm pore size; Millipore, USA) covered with or without Matrigel were used. SW480 and HCT116 cells (2 × 10^5^ cells) with the medium (serum-free) were inoculated in the upper chamber. A complete medium was added into the bottom chamber. The cell plate was then incubated at 37°C for 24 h. After that, the filter’s top cells were removed by cotton swabs. The cells that had traveled to the membrane’s bottom were fixed, and then exposed to crystal violet (0.2%) for 5 min. An inverted microscope was used to measure migration and invasion cell numbers.

### Immunofluorescence Assay

An immunofluorescence assay was utilized to detect expression levels of CD206 and CD86, which are biomarkers of M1 and M2 macrophages. THP-1 cells were cultivated to 70% confluence on the glass side. Then, cells were fixed with paraformaldehyde (4%) for 1 h after washing twice with 1 × PBS. Next, cells were permeabilized with Triton X-100 (0.2% ) in 1 × PBS at 37°C for 15 min and closed by normal goat serum (10%) at 25°C for 1 h. Subsequently, cells were incubated with primary antibodies (Abcam), including anti-CD86 antibody (ab239075, dilution: 1/100), anti-CD206 antibody (ab300621, dilution: 1/50), anti-CD31 antibody (ab76533, dilution: 1/500), and anti-CKAP2 antibody (ab227214, dilution: 1/1000) for 1 day at 4°C. Then, cells were incubated with the goat anti-rabbit secondary antibody (ab207995, Abcam, dilution: 1/1000). Ultimately, cells were stained with DAPI and a microscope was used to observe them.

### Enzyme-Linked Immunosorbent Assay (ELISA)

THP-1 cells (1 × 10^5^) re-suspended with complete medium (300 μl) were plated in 96-well plates, followed by the collection of the supernatant. IL-1β and CCL17 concentrations in cell culture supernatants were measured via a human IL-1β ELISA kit and a human CCL17 ELISA kit (R&D Systems, China), respectively.

### Tube Formation Assay

HUVECs (2 × 10^4^) were put in the 96-well plate coated with Matrigel, and then the nc-HCT116/THP-1 and oe-HCT116/THP-1 supernatants were added. The HUVEC tubular structure was observed under the inverted microscope after cultivating for 8 h.

### Dual-Luciferase Reporter (DLR) Assay

In examining the relative luciferase activity, we applied a DLR assay to confirm the TFDP1 and CKAP2 interaction. To generate pGL3-CKAP2 promoter-wild type (WT), pGL3-CKAP2 promoter-mutated (MUT) 1/2 luciferase reporters, WT CKAP2 and MUT1/2 CKAP2 sequences were introduced into pGL3-basic (Promega, USA). After that, the pcDNA3.1-NC or pcDNA3.1-TFDP1 was co-transfected with pGL3-CKAP2 promoter-WT or pGL3-CKAP2 promoter-MUT1/2 luciferase reporter in the SW480 and HCT116 cells for 2 days.

### Co-Immunoprecipitation Assay (Co-IP)

We used Co-IP assay to confirm the interaction between CKAP2 and TFDP1. First, HCT116 and SW480 cells were lysed in RIPA buffer (50 mM Tris (pH 7.4), 150 mM NaCl, 1 mM EDTA, 1% Triton X-100, 10% glycerol, 10 μg/ml leupeptin, 10 μg/ml aprotinin, and 2 mM PMSF) for 4 h, and then centrifuged (12,000 ×*g*, 10min). After that, cell supernatants were immunoprecipitated with the anti-CKAP2 antibody (ab85889, Abcam) and incubated with magnetic protein A/G beads at 4°C for 2 h. Finally, for gel electrophoresis, we re-suspended precipitated beads in 2 × loading buffer.

### Mice Tumorigenesis Assay

Male nude mice (BALB/c, 5 weeks of age) were bought from Vital River Laboratories (China). Mice were routinely housed for one week for adaption to the environment. HCT116 cells (1 × 10^6^) transfected with sh-CKAP2/sh-NC were injected (subcutaneously) into the nude mice flanks. Subcutaneous tumor size was determined every 7 days during the 7-35 day period. Tumor volume was calculated based on the given formula: V = (shortest diameter)^2^ × (longest diameter) × 0.5. After 4 weeks, the mice were sacrificed using a pentobarbital overdose. Permission for each experiment was given by the Animal Ethics Committee of Beijing Viewsolid Biotechnology Co., Ltd. (VS2126A00163). All methods are reported following the ARRIVE guidelines.

Each mouse was injected with luciferase-labeled HCT116 cells (1 × 10^6^) transfected with sh-NC/sh-CKAP2 via the tail vein. After 60 days, the IVIS was used to observe lung metastasis of mouse tumors.

### Immunohistochemistry (IHC) Staining

IHC staining was performed to assess Ki-67 and CKAP2 protein expression in tumor tissues, which were fixed in paraformaldehyde (4%), embedded in paraffin, and sliced (5-μm). Tissue segments were incubated with antibodies (Abcam), including anti-Ki-67 (ab92742, dilution: 1:200), anti-CKAP2 (ab198188, dilution: 1:200), anti-CD163 (ab182422, dilution: 1:500), and anti-CD31 (ab182981, dilution: 1:2000) overnight. Next, tissue sections were incubated with goat anti-rabbit secondary antibody (ab6721, dilution/1:1000) for 2 h, and then nuclear counterstained with hematoxylin. Finally, positively stained cells were assessed.

### Hematoxylin and Eosin (HE) Staining

The metastasis of mice CRC cells to lung tissues was evaluated by using the HE staining method. Lung tissues of mice were embedded in paraffin and then cut into segments (5 μm) which were then stained with HE. Finally, sections were dehydrated with anhydrous alcohol, sealed with a neutral sealant, and examined under a microscope.

### Data Analysis

Data analysis was performed using GraphPad Prism 7.0. Data were displayed using the mean ± SD. Group differences among multiple groups were analyzed using a one-way ANOVA. Comparisons between two groups were conducted through a Student’s *t*-test. *p* < 0.05 was regarded as a significant value.

## Results

### High Expression Levels of CKAP2 in CRC Cells and Tissues

**Fig. 1:**
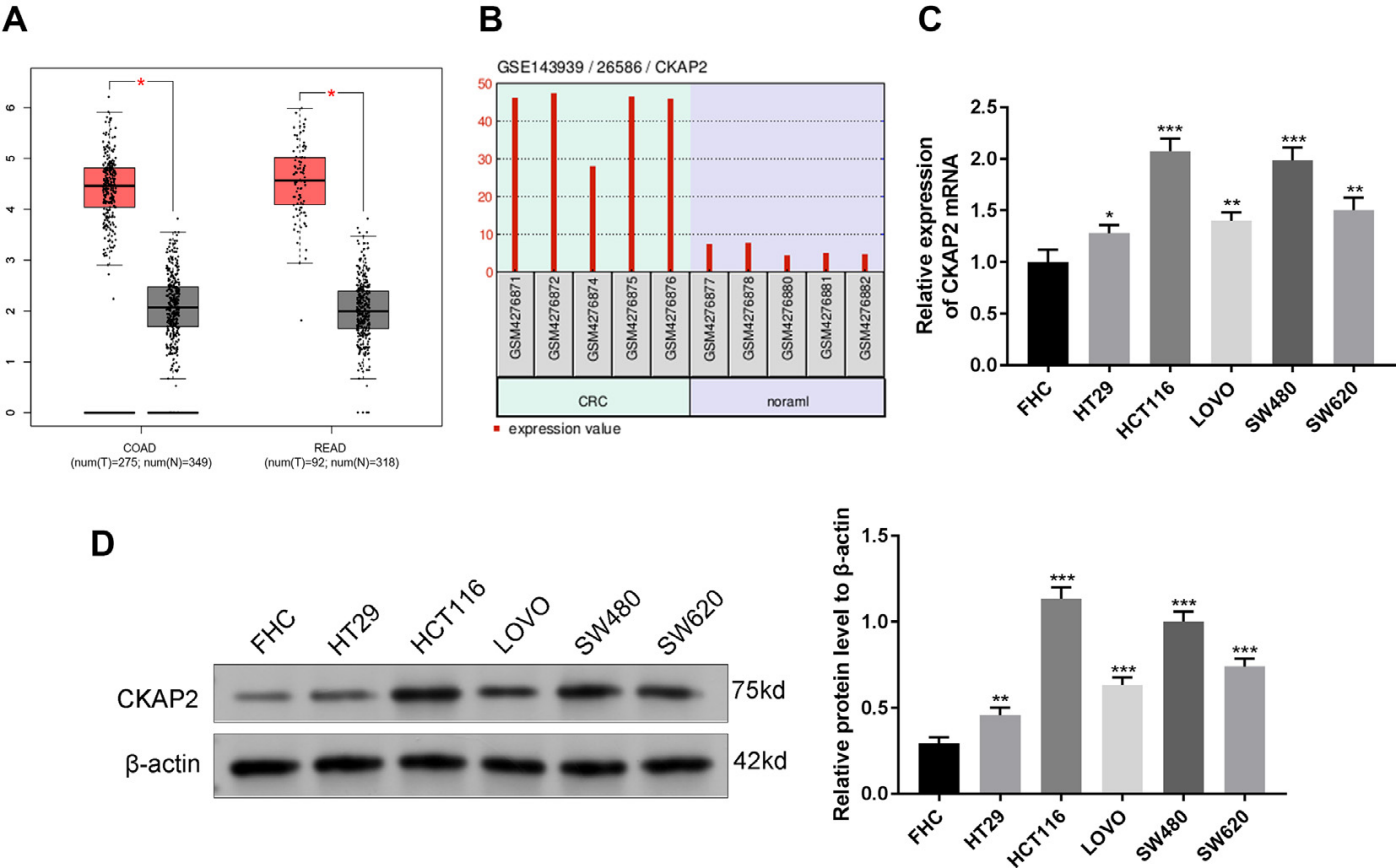
CKAP2 expression level is boosted in CRC. (**A**) Analyzing CKAP2 expression in CRC using the GEPIA database (TCGA-COAD and READ). (**B**) Analyzing CKAP2 expression level in 5 pairs of CRC tissues and adjacent tissues in the GEO database GSE143939. (**C**) Detection of CKAP2 mRNA expression level in CRC cell lines and a normal human colon epithelial cell line (**FHC**) by qRT-PCR. (**D**) Detection of CKAP2 protein expression in CRC cell lines and FHC cells by western blotting. ***p < 0.001, **p < 0.01, *p < 0.05. We executed each experiment three times.

The Cancer Genome Atlas (TCGA)-colon adenocarcinoma (COAD) and TCGA-rectum adenocarcinoma (READ) were selected for analysis through Gene Expression Profiling Interactive Analysis (GEPIA). CKAP2 was highly expressed in COAD tissues and READ tissues compared to normal tissues (Fig. 1A, *p* < 0.05). A dataset (GSE14359, consisting of 5 pairs of adjacent tissues and colon cancer) in the GEO database revealed significant upregulation of CKAP2 expression in CRC (Fig. 1B). Moreover, the mRNA expression level of CKAP2 in CRC cell lines (HCT116, SW480, HT29, LOVO, and SW620) was higher than that in the FHC cell line (Fig. 1C, *p* < 0.05). The western blotting result revealed that CKAP2 expression was significantly increased in CRC cells (HT29, HCT116, LOVO, SW480, SW620) comparing to FHC cells, especially in SW480 and HCT116 cells (Fig. 1D, *p* < 0.01).

### Silencing of CKAP2 Represses Migration, Proliferation, Invasion, and EMT of CRC Cells

**Fig. 2:**
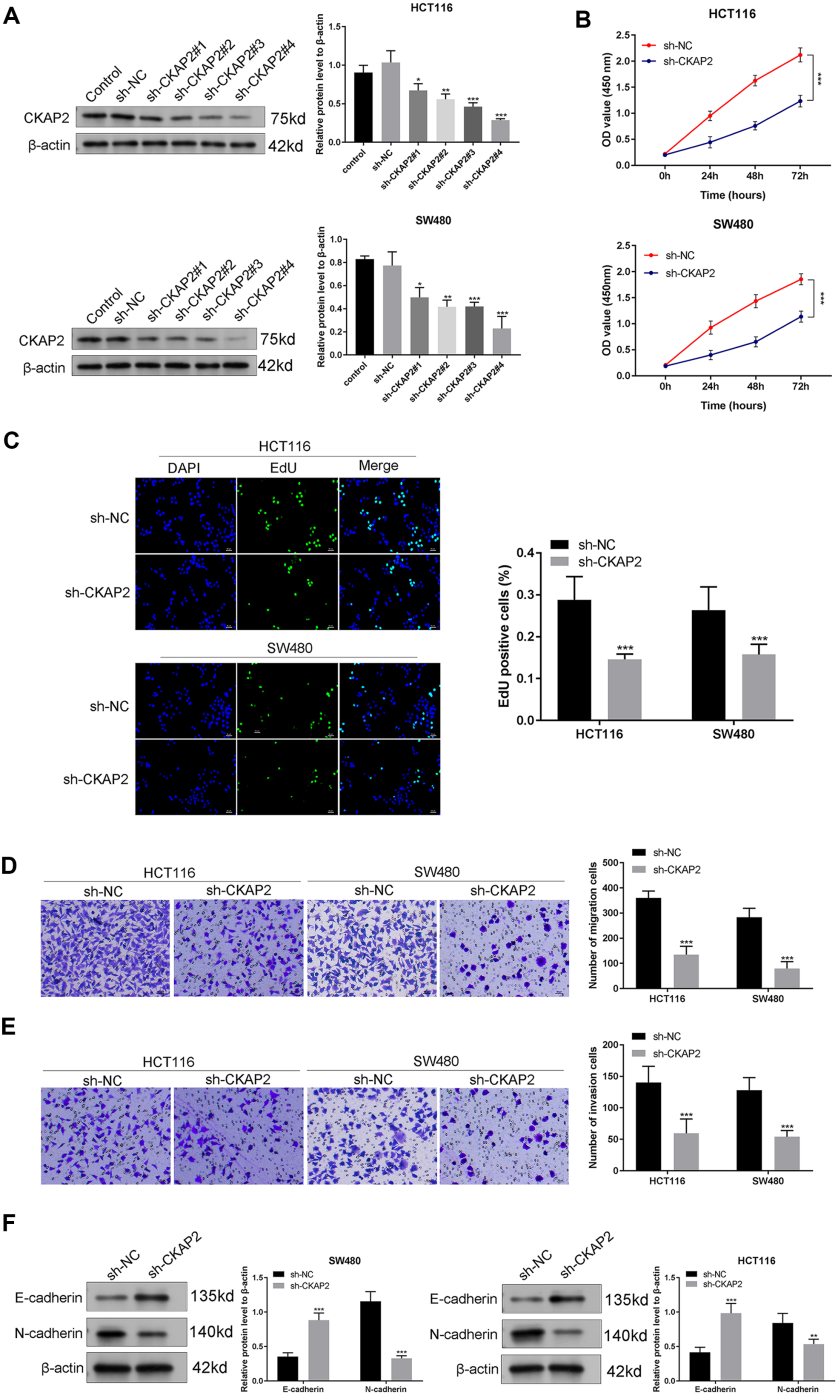
Silencing of CKAP2 prevents proliferation, migration, invasion, and EMT of CRC cells. (**A**) Western blotting could detect the knockout efficiency of CKAP2 in HCT116 and SW480 cells. (**B**) The cell viabilities of HCT116 and SW480 cells were evaluated by CCK-8 assay. (**C**) HCT116 and SW480 cell proliferation was assessed by EdU assay. (**D**) Transwell assay could detect the migration ability of HCT116 and SW480 cells. (**E**) Transwell assay was employed to detect the invasion ability of HCT116 and SW480 cells. (**F**) Western blotting was utilized for detecting E-cadherin and N-cadherin protein levels in HCT116 and SW480 cells. ***p < 0.001, **p < 0.01, *p < 0.05. Each experiment was executed in triplicate.

To explore the CKAP2 function, CKAP2 was silenced in SW480 and HCT116 cells. According to western blotting, sh-CKAP2#1, #2, #3, and #4 could effectively reduce CKAP2 protein levels compared to sh-NC in SW480 and HCT116 cell lines (Fig. 2A, *p* < 0.05). Among them, sh-CKAP2#4 had the most significant knockout effect and was selected for subsequent experiments. CCK-8 assay demonstrated that HCT116 and SW480 cells’ viability was markedly attenuated after silencing of CKAP2 (Fig. 2B, *p* < 0.001). The EdU assay suggested that HCT116 and SW480 cell proliferation was evidently inhibited after silencing of CKAP2 (Fig. 2C, *p* < 0.001). The transwell assay indicated that silencing of CKAP2 impaired the invasion and migration of SW480 and HCT116 cells (Fig. 2D, 2E, *p* < 0.001). In addition, CKAP2 downregulation resulted in the increase of E-cadherin expression and the decrease of N-cadherin expression in HCT116 and SW480 cells (Fig. 2F, *p* < 0.01).

### CKAP2 Affects the Differentiation of TAMs in Tumor Microenvironment

**Fig. 3:**
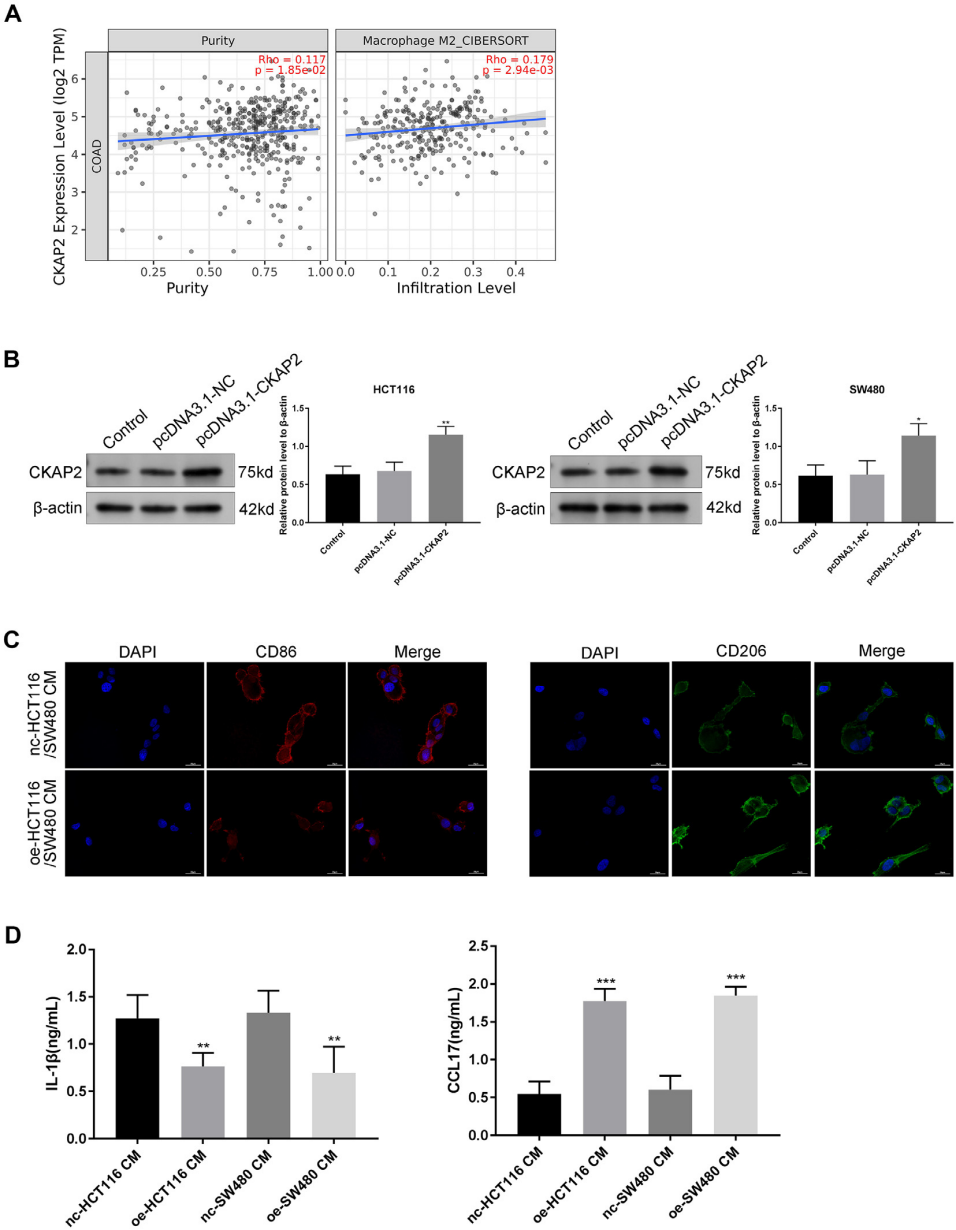
CKAP2 affects the differentiation of TAMs in the tumor microenvironment. (**A**) The relationship between CKAP2 and M2 macrophages was predicted through the Timer2.0 database. (**B**) Western blotting could detect the overexpression efficiency of CKAP2 in CRC cells. (**C**) Immunofluorescence was used to detect CD86 and CD206 in THP-1 cells. (**D**) ELISA could detect the concentration of IL-1β (M1 macrophage-related cytokine) and CCL17 (M2 macrophagesrelated cytokine) in THP-1 supernatant. ***p < 0.001, **p < 0.01, *p < 0.05. We executed each experiment three times.

We first discovered a substantial relationship between CKAP2 and M2 macrophages through Timer2.0 (Fig. 3A). Then, we transfected pcDNA3.1-NC and pcDNA3.1-CKAP2 into SW480 and HCT116 cells (Fig. 3B, *p* < 0.05). Next, we collected the supernatant of SW480 and HCT116 cells that were transfected with pcDNA3.1-NC or pcDNA3.1-CKAP2 (nc-HCT116/SW480 CM or oe-HCT116/SW480 CM) to treat PMA-induced THP-1 cells. Immunofluorescence was utilized for detecting the expression levels of CD86 (a protein marker of M1-polarized macrophages) and CD206 (a protein marker of M2-polarized macrophages) in THP-1 cells. Compared to THP-1 cells treated with nc-HCT116/SW480 CM, THP-1 cells treated with oe-HCT116/SW480 CM had fewer M1 macrophages, but more M2 macrophages (Fig. 3C). The ELISA results confirmed a decreased concentration of IL-1β (M1 macrophages-related cytokine) in the THP-1 cell supernatant after CKAP2 overexpression, and an increased CCL17 concentration in the THP-1 cell supernatant after CKAP2 overexpression. This result suggested the promoting effect of CKAP2 on M2 macrophage polarization (Fig. 3D, *p* < 0.01).

### CKAP2 Promotes HUVEC Proliferation and Angiogenesis by Influencing the Tumor Microenvironment

**Fig. 4:**
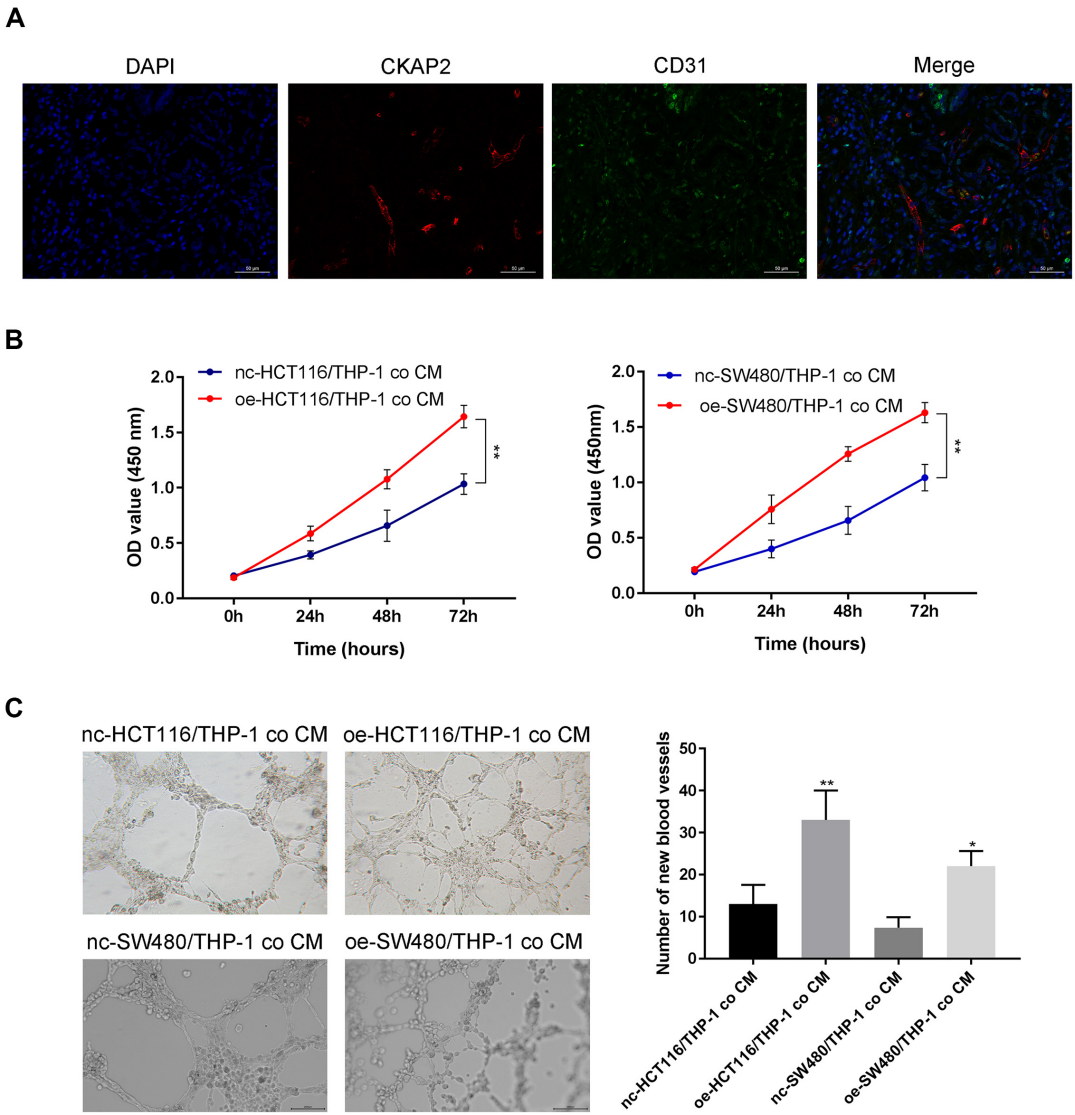
CKAP2 facilitates the proliferation of HUVECs and angiogenesis by influencing the tumor microenvironment. (**A**) Protein expression levels of CKAP2 and CD31 (microvascular marker) were stained in CRC tissues by immunofluorescence. Cancer/THP-1 co CM was constructed by co-culturing HCT116/SW480 cells transfected with pcDNA3.1-NC/pcDNA3.1-CKAP2 and THP-1 cells in a 6-well plate for just 48 h, centrifuging to eliminate debris, and HCT116/SW480/THP-1 co CM was then obtained. The HCT116/SW480/THP-1 co CM was used to culture HUVECs. (**B**) CCK-8 assay was used to detect the proliferation of HUVECs. (**C**) Tube formation assay could detect the angiogenesis of HUVECs. ***p < 0.001, **p < 0.01, *p < 0.05. We executed each experiment three times.

According to the immunofluorescence assay, CKAP2 and CD31 (a microvascular marker) were co-expressed in CRC tissues (Fig. 4A). THP-1 cells were co-cultured with HCT116/SW480 cells transfected with pcDNA3.1-NC/pcDNA3.1-CKAP2 for 48 h, and the supernatant was obtained by centrifugation to culture HUVECs. The CCK-8 results confirmed that HUVECs cultured by oe-HCT116/THP-1 co CM and oe-SW480/THP-1 co CM had stronger proliferation ability than the HUVECs cultured by nc-HCT116/THP-1 co CM and nc-SW480/THP-1 co CM (Fig. 4B, *p* < 0.01). oe-HCT116/THP-1 co CM and oe-SW480/THP-1 co CM enhanced the ability of HUVECs to generate blood vessels in a tube formation assay (Fig. 4C, *p* < 0.05).

### TFDP1 Regulates CKAP2 Expression

**Fig. 5:**
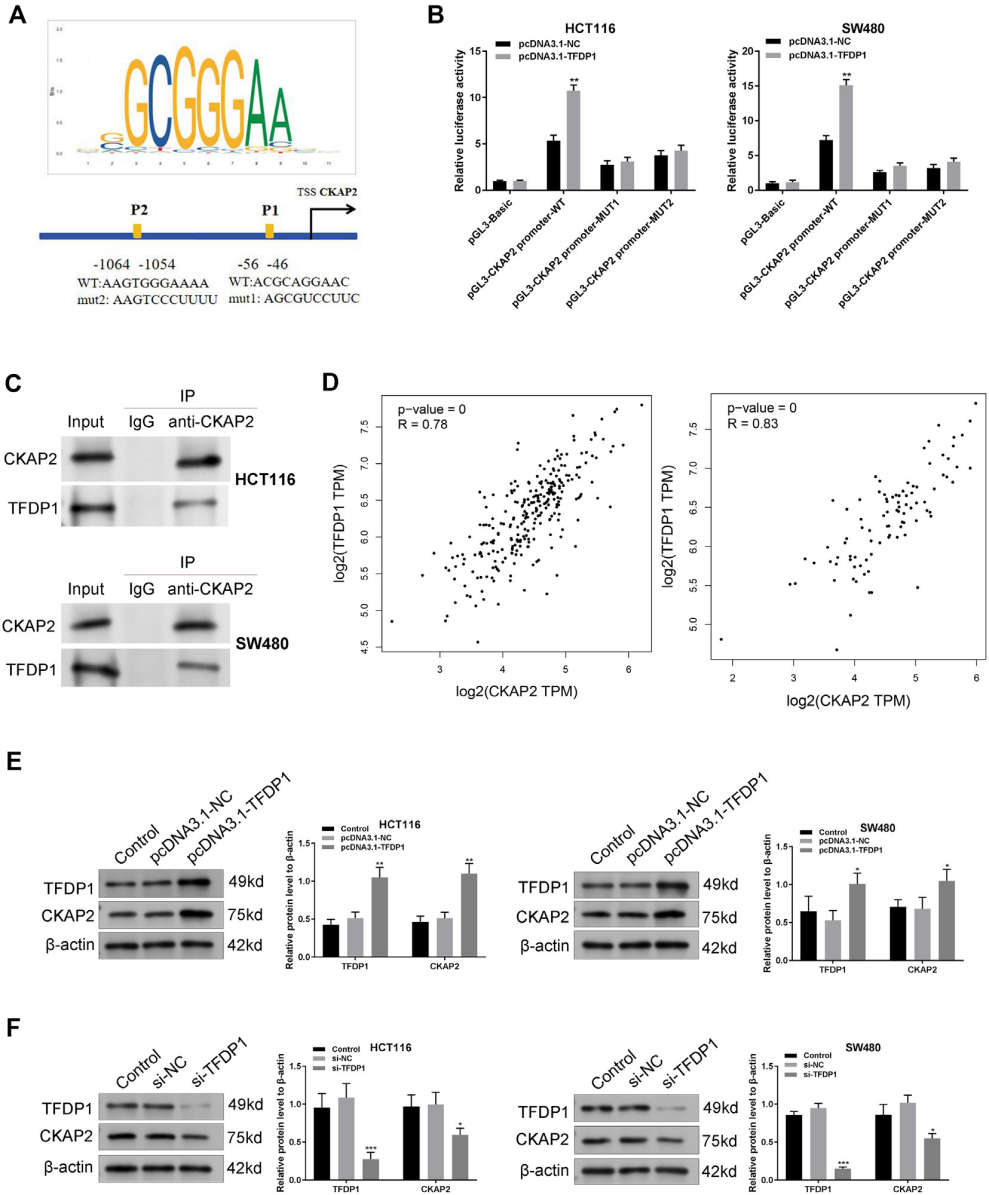
TFDP1 directly regulates CKAP2 expression. (**A**) Online prediction of binding sites between TFDP1 and CKAP2 promoter sequences through JASPAR (https://jaspar.genereg.net/). (**B, C**) The interaction between TFDP1 and CKAP2 in HCT116 and SW480 cells was confirmed using dual luciferase reporter assay and co-immunoprecipitation assay. (**D**) GEPIA online analysis of the correlation between the TFDP1 and CKAP2 expression levels in COAD (Colon adenocarcinoma) and READ (Rectum adenocarcinoma) databases. (**E**) Western blotting could detect protein levels of TFDP1 and CKAP2 in HCT116 and SW480 cells. (**F**) Western blotting could detect protein levels of TFDP1 and CKAP2 in HCT116 and SW480 cells. *p < 0.05, **p < 0.01, ***p < 0.001. Each experiment was executed in triplicate.

Online prediction in the JASPAR database predicted binding sites between TFDP1 and CKAP2 promoter sequences (Fig. 5A). To verify the relationship between TFDP1 and CKAP2, a DLR assay was performed. We discovered that overexpression of TFDP1 increased fluorescence intensity in pGL3-CKAP2 promoter-WT groups. However, in the pGL3-CKAP2 promoter MUT1/2 groups, overexpression of TFDP1 could not affect fluorescence intensity (Fig. 5B, *p* < 0.01). Also, the result of the Co-IP assay revealed specific enrichment of TFDP1 co-precipitated within CKAP2 immunocomplex in HCT116 and SW480 cells (Fig. 5C), suggesting the interaction between CKAP2 and TFDP1. Moreover, online analysis of the GEPIA database revealed a positive relationship between the expressions of TFDP1 and CKAP2 in COAD and READ (Fig. 5D). TFDP1 and CKAP2 protein levels in SW480 and HCT116 cells were increased by TFDP1 overexpression (Fig. 5E, *p* < 0.05). The TFDP1 and CKAP2 expression levels were reduced by TFDP1 downregulation in SW480 and HCT116 cells (Fig. 5F, *p* < 0.05).

### Overexpression of CKAP2 Partially Reverses the Effects of TFDP1 Downregulation on Proliferation, Migration, and Invasion of CRC Cells

**Fig. 6:**
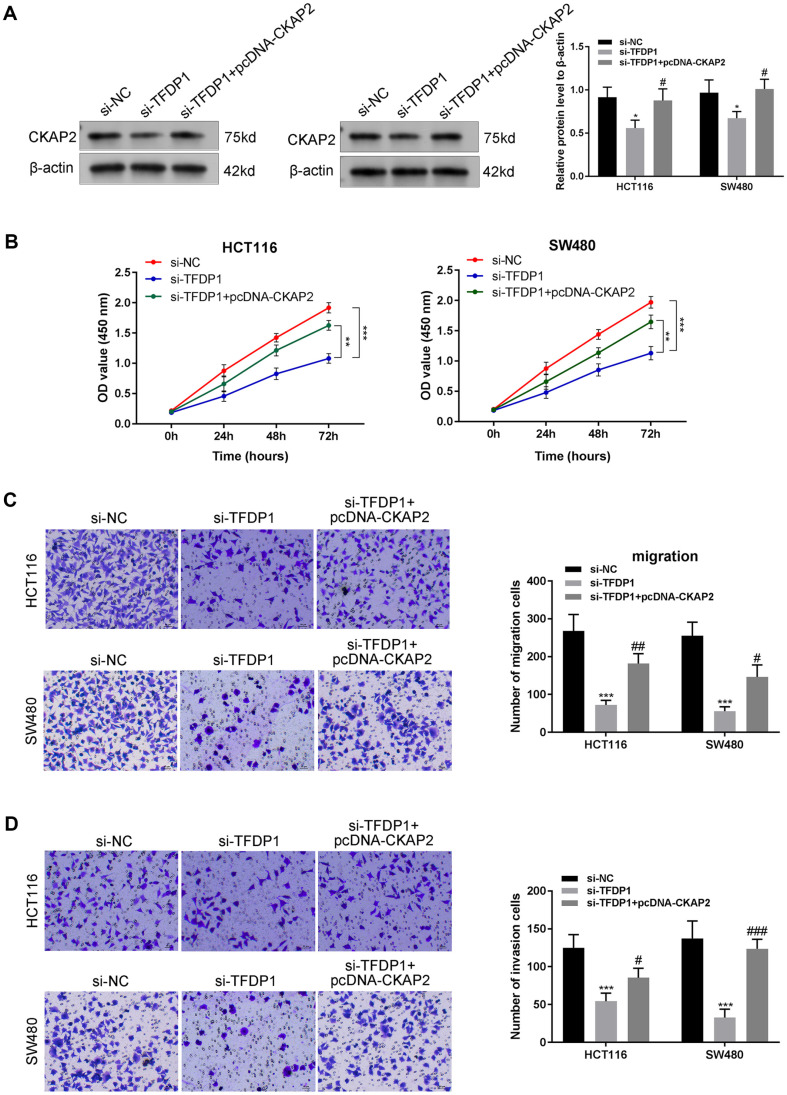
Overexpression of CKAP2 partially reverses the effect of TFDP1 downregulation on CRC cells. (**A**) The CKAP2 protein level in different groups (si-NC, si-TFDP1, si-TFDP1+pcDNA-CKAP2) of HCT116 and SW480 cells was detected by western blotting. (**B**) The proliferation of HCT116 and SW480 cells was assessed by EdU assay. (**C**) Transwell assay was conducted to detect the migration ability of HCT116 and SW480 cells. (**D**) Transwell assay was conducted to detect the invasion ability of HCT116 and SW480 cells. *p < 0.05, **p < 0.01, ***p < 0.001. ^#^*p* < 0.05, ^##^*p* < 0.01, ^###^*p* < 0.001. Each experiment was executed in triplicate.

Next, we explored whether CKAP2 affected proliferation, migration, and invasion via interacting with TFDP1 in CRC cells. We found that the protein level of CKAP2 in SW480 and HCT116 cells was reduced after TFDP1 downregulation, which was reversed by CKAP2 overexpression (Fig. 6A, *p* < 0.05). Based on the CCK-8 assay, the proliferation abilities of SW480 and HCT116 cells were significantly inhibited by TFDP1 downregulation, and the inhibitory effect of TFDP1 downregulation on cell proliferation was reversed by CKAP2 overexpression (Fig. 6B, *p* < 0.01). Also, HCT116 and SW480 cell migration and invasion were suppressed by TFDP1 downregulation, and the suppressive effects of TFDP1 downregulation on cell migration and invasion were reversed by CKAP2 overexpression (Fig. 6C, 6D, *p* < 0.05).

### CKAP2 Downregulation Impairs Tumor Growth and Metastasis In Vivo

**Fig. 7:**
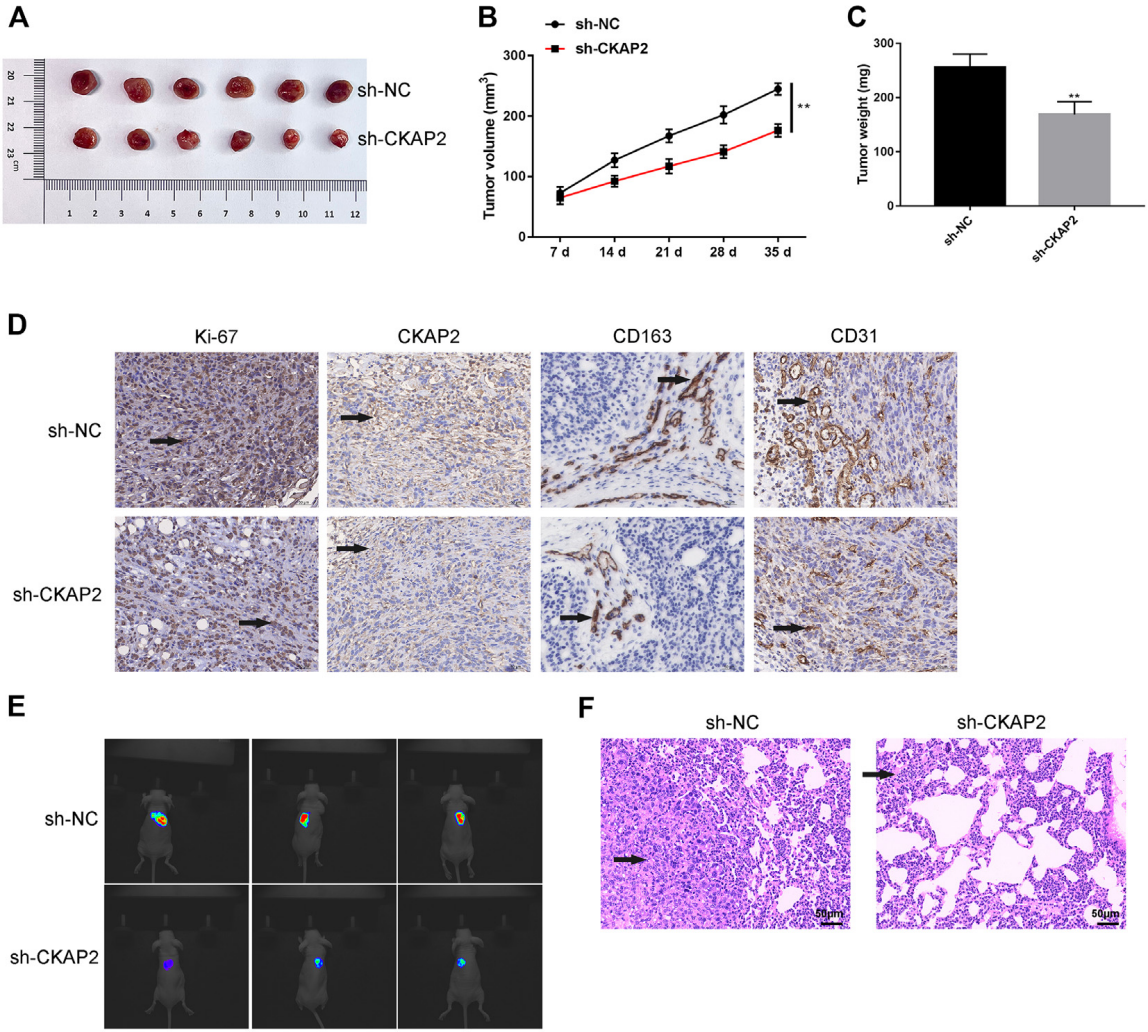
CKAP2 downregulation inhibits in vivo tumor growth and metastasis. (**A**) HCT116 cells were inoculated in nude mice, and the size of the subcutaneous tumor was measured every 7 days during the 7-35 day period. (**B, C**) Tumor volume and tumor weight were displayed. (**D**) Positive staining of Ki-67, CKAP2, CD163, and CD31 in tumor tissues was detected by IHC staining. (**E**) The IVIS was used to monitor tumor lung metastasis in mice. (**F**) The lung tissues of nude mice were used for HE staining to observe lung metastasis of CRC. **p < 0.01.

Finally, we confirmed the role of CKAP2 in CRC in vivo. The subcutaneous tumor formation assay in nude mice showed that CKAP2 silencing significantly reduced the size of mouse tumors (Fig. 7A), as well as tumor volume and tumor weight (Fig. 7B, 7C, *p* < 0.01). We analyzed the experimental results of IHC and observed that the positive staining (brown area) of Ki-67, CKAP2, CD163, and CD31 was reduced after CKAP2 knockdown in the tumor tissues of mice, showing the inhibitory effects of CKAP2 knockdown on proliferation and angiogenesis of tumor cells (Fig. 7D). Through IVIS, we found that CKAP2 downregulation resulted in the decrease of luciferase-labeled tumor cells in mice, suggesting the inhibitory effect on tumor lung metastasis in vivo (Fig. 7E). Meanwhile, HE staining of mice lung tissues revealed the reduction in tumor nodules after CKAP2 downregulation (Fig. 7F).

## Discussion

The potential pathogenesis of CRC, a highly lethal malignant tumor, is increasingly being explored to develop new therapeutic drugs [25-27]. The decrease in expression level in MIIP leads to excessive AZGP1 secretion, resulting in poor prognosis and rapid progression of CRC induced by fat browning [27]. There is also evidence suggesting that decreased iron intake and low systemic iron levels are related to the pathogenesis of CRC [25, 28]. To find more accurate and effective diagnostic and treatment methods for CRC, we have searched for more possible targeted genes and investigated their pathogenic mechanisms based on existing research. We found high CKAP2 expression in CRC cells and tissues, and silencing of CKAP2 could prevent CRC cells from proliferating, migrating, invading, and EMT. We inferred that CKAP2 functions as an oncogene in CRC. Moreover, we constructed a nude mouse model of CRC and found that silencing of CKAP2 suppressed metastasis and tumor growth in nude mice.

TAMs have become recognized as the most abundant stromal cells in many tumor microenvironments in recent years [29-32]. Macrophages participate in inflammation and immunity and have a wide range of biological activities, but these biological activities often have the opposite characteristics [33]. The M2 macrophages of TAMs promote inflammation and tumor progression [34]. We found a significant correlation between CKAP2 and M2 macrophages through bioinformatics analysis. Cervical cancer conditioned medium affects macrophage differentiation and leads to cervical cancer [32]. Therefore, we used the supernatant of CRC cells as a conditioned medium to culture THP-1 cells and found that overexpressing CKAP2 in the conditioned medium led to more differentiation of THP-1 cells into M2 macrophages. In addition, we also found that CKAP2 promoted the angiogenesis and proliferation of HUVECs by influencing the tumor microenvironment. Liu *et al*.'s research also suggests that TAMs can maintain the tumor immune suppressive microenvironment, promote angiogenesis, and facilitate tumor cell metastasis [35]. Angiogenesis provides abundant nutrients for tumor growth; therefore, anti-angiogenic therapy plays an important role in tumor treatment.

Transcription factors, as transcription initiation elements, are crucial for gene expression [36]. We found binding sites between TFDP1 and CKAP2 promoter sequences through online prediction in the JASPAR database. TFDP1 exerts a crucial function in various important life processes by interacting with E2F1 [37, 38]. Thus, we speculated that tumor progression caused by overexpression of CKAP2 is related to TFDP1. Our research results also displayed a positive relationship between TFDP1 and CKAP2 expression levels in CRC cells and tissues. Expression of CKAP2 could reverse the impacts of TFDP1 downregulation on migration, invasion, and proliferation of CRC cells.

We found highly expressed and regulated CKAP2 in CRC by TFDP1. CKAP2 endorses the proliferation and metastasis of CRC via moving macrophage differentiation in the tumor microenvironment. Our research provides new biological targets for managing CRC.

## Figures and Tables

**Table 1:** Sequences of PCR primers.

Gene	Forward primer	Reverse primer
CKAP2	5’-CGGCCTTCCGAGAACAAAGA-3’	5’-TGGACCCGATCCTCAGATGT-3’
GAPDH	5’-CTCTGATTTGGTCGTATTGGGC-3’	5’-CCTGGAAGATGGTGATGGGATT-3’
